# Promoting post-traumatic growth in cancer patients: a randomized controlled trial of guided written disclosure

**DOI:** 10.3389/fpsyg.2024.1285998

**Published:** 2024-03-28

**Authors:** Valentina Cafaro, Elisa Rabitti, Giovanna Artioli, Massimo Costantini, Francesco De Vincenzo, Francesca Franzoni, Silvio Cavuto, Tatiana Bertelli, Giuseppe Deledda, Angela Piattelli, Lisa Cardinali, Silvia De Padova, Sara Poli, Maria Domenica Iuvaro, Giovanna Fantoni, Silvia Di Leo

**Affiliations:** ^1^Psycho-oncology Unit, Azienda USL-IRCCS di Reggio Emilia, Reggio Emilia, Italy; ^2^Rete Cure Palliative Dipartimento Cure Primarie, Azienda USL-IRCCS di Reggio Emilia, Reggio Emilia, Italy; ^3^Department of Medicine and Surgery, University of Parma, Parma, Italy; ^4^Scientific Directorate, Fondazione Istituto di Ricovero e Cura a Carattere Scientifico (IRCCS) Istituto Nazionale dei Tumori di Milano, Milan, Italy; ^5^Department of Human Sciences, European University of Rome, Rome, Italy; ^6^Clinical Trials and Statistics Unit, SOC Infrastructure, Research and Statistics, Azienda USL-IRCCS di Reggio Emilia, Reggio Emilia, Italy; ^7^Psycho-oncology Service, Palliative Care, Pain Therapy and Integrative Medicine Unit, IRCCS Istituto Romagnolo per lo Studio dei Tumori (IRST) “Dino Amadori”, Meldola, Italy; ^8^Unit of Clinical Psychology, IRCCS Hospital Sacro Cuore Don Calabria, Negrar di Valpolicella (Verona), Verona, Italy; ^9^UOC Oncologia Medica - Dipartimento Oncoematologico Azienda Ospedaliera di Cosenza, Cosenza, Italy

**Keywords:** post-traumatic growth, guided written disclosure, RCT, cancer, meaning making, writing intervention, post-traumatic stress

## Abstract

**Background:**

Cancer is a life-threatening disease that triggers not only vulnerability to distressing symptoms but also a meaning-making process that may lead to post-traumatic growth. As people often struggle to integrate cancer illness into their meaning system to reach an adaptive meaning, psychological interventions focused on a reappraisal of the illness experience can facilitate this process. This multicenter randomized controlled trial (RCT) was primarily aimed at assessing the efficacy of a writing intervention known as a guided disclosure protocol (GDP), compared to a generic writing intervention, in promoting post-traumatic growth in stage I-III breast and colon cancer patients at the end of their adjuvant chemotherapy.

**Methods:**

Between January 2016 and August 2020 recruitment of eligible subjects took place during follow-up clinical consultations. Assessment occurred at baseline (T0), after the intervention (T1, 3 months from baseline), and at 6 months from baseline (T2). Both interventions consisted of three 20-min writing sessions to be performed once every two weeks. Change in post-traumatic growth was assessed using the Posttraumatic Growth Inventory, meaning with the Constructed Meaning Scale, and psychological distress with the Impact of Event Scale and the Hospital Anxiety and Depression Scale.

**Results:**

In the five participating centers, 102 eligible patients were randomized and 72 participants completed follow-up evaluation. Most patients were women (79.4%), had breast cancer (68.6%), and stage I (27.5%), or stage II (44.1%) disease. Mean differences did not reach statistical significance for any of the dependent variables. However, an effect of the GDP, although not statistically relevant, was observed after the intervention in terms of enhanced post-traumatic growth and increased distress measured with the Impact of Event Scale.

**Conclusion:**

This is the first RCT investigating the efficacy of a GDP in cancer patients having post-traumatic growth as the primary aim. Though GDP is a promising intervention in promoting post-traumatic growth, the lack of statistical significance of our findings may be due to the study being underpowered, hence this trial should be replicated with an adequate sample size, paying attention to supporting recruitment.

Clinical trial registration: ClinicalTrials.gov, identifier: 2015/0024360.

## 1 Introduction

Being affected by cancer is one of the most distressing occurrences that human beings can encounter in their lives. Patients often face cultural stigma and social isolation, and in addition, aggressive and iatrogenic treatments may have both physical and psychosocial sequelae, negatively impacting patients’ quality of life (Foster et al., 2009). Even in the case of patients with early-stage disease, the risk of recurrence is frequently perceived as a threat. Consequently, cancer increases individual vulnerability to post-traumatic stress symptoms, depression, anxiety, and intrusive thoughts (Koopman et al., 2002; Christensen et al., 2009).

However, dysfunctional symptoms are not the only possible outcomes occurring after a cancer diagnosis. In the last two decades, a growing body of literature has also investigated the potential benefits of stressful events, focusing on the role that meaning plays in people’s lives (e.g., Davis et al., 1998; Morasso et al., 2008; Wang et al., 2015).

Park (2010) has proposed a meaning-making model describing how people make sense of the world through a global meaning system, allowing them to perceive a coherent world and to orient their lives shaping individuals’ thoughts, emotions, and actions. The feeling of meaningfulness includes a sense of purpose that self-perpetuates through behaviors that individuals enact with the aim of reaching their desired goals. It is assumed that this is constructed early in life and then adjusted according to experiences.

Park’s (2010) meaning-making model also seems to be helpful in revealing the process triggered when a traumatic experience, such as cancer illness can be, negatively affects one’s life. Since such a shattering experience cannot be easily integrated into the person’s global meaning system, it may trigger a new search for meaning in order to overcome the meaning discrepancy. A discrepancy between the meaning attributed to a traumatic event (i.e., appraised meaning) and the person’s global beliefs and orienting systems (i.e., global meaning) generates indeed distress, which prompts meaning-making efforts. In other words, it concerns how people dealing with a life-threatening illness react and behave to adjust to the novel condition, finding a new sense to the world and to their personal identity within this world (Fife, 2005). Discrepancy reduction can happen through assimilation or accommodation. According to the former, the appraised meaning of the traumatic event changes so that it is seen as less problematic or aversive; according to the latter, the global meaning changes, therefore beliefs and goals are modified and priorities are reordered (Park and George, 2013). The meaning-making process seems to be closely connected to the possibility of perceiving personal growth following a traumatic experience.

Post-traumatic growth (PTG) concerns all the positive changes that people experience as a result of having struggled with a traumatic event (Park et al., 2010). According to the inventory developed by Tedeschi and Calhoun, PTG includes the following factors: New Possibilities, Relating to Others, Personal Strength, Spiritual Change, and Appreciation of Life (Tedeschi and Calhoun, 1996). Although PTG is becoming a clear and well-established construct, the cognitive process and the narrative development underlying this process are still not clear (Tedeschi and Calhoun, 2004).

The relationship between PTG and the meaning-making process has been investigated by several authors in different populations. For instance, Groleau et al. (2013) highlight how students with a traumatic background, who managed to find meaning in their stressful experience, had higher levels of PTG compared with students who did not. In a qualitative study, head and neck cancer survivors described the occurrence of the illness related trauma and distress as a catalyst for finding new meaning in life through deep reflection and redefinition of life priorities to achieve a greater life satisfaction and fulfillment (Threader and McCormack, 2016). Nevertheless, the methodological structure of these studies limits the understanding of the nature of the relationship between the two constructs. As a consequence, no clear evidence exists explaining the mechanisms connecting meaning and PTG (Aliche et al., 2022). According to two recent cross-sectional studies, presence of meaning and gender are the strongest predictors of PTG in people with a chronic illness (Zeligman et al., 2018), and meaning making and the search for meaning are predictive of PTG in trauma survivor students (Zeligman et al., 2019). With reference to studies performed on cancer patients, Li et al. (2015) suggest that PTG appears correlated to high sense-making and benefit finding, and also that high PTG cancer patients experience less demoralization. Wang et al. (2015) report that, consistently with the meaning-making model, a higher level of discrepancy in meaning was significantly related with greater meaning making. Moreover, the relationship between meaning making and PTG is mediated by a high level of construal, i.e., meaning making elicits a cognitive strategy through which people transcend the situational-dependent view to adopt a broader and more comprehensive perspective.

Integrating a traumatic experience within the individual meaning system can be a difficult task in the absence of the psychological resources leading to PTG (Neimeyer, 2006), and cancer patients may experience a high level of depression and anxiety (Li et al., 2015).

The hypothesis that a traumatic experience introduces information discrepant with the individual’s global meaning system is not a novelty as post-traumatic stress disorder (PTSD) theories highlight the need for modifying pre-existing schemas to integrate new information (Horowitz, 1990; Park et al., 2012). Research on neurological correlates of PTG have supported this hypothesis, and authors have proposed that the activation of brain area reflecting approach-oriented strategies might be central for the active engagement in new goals and possibilities and, consequently, in new and different schemas (Rabe et al., 2006). Moreover, PTG has been shown to be correlated to a greater association between the brain areas liable for memory and mentalization; these functions support the social abilities that are crucial to experience enhanced relationships, which is one of the PTG dimensions.

The relationship between PTG and post-traumatic stress (PTS) has been the object of theorizations and empirical research. Tedeschi and Calhoun (2004) hypothesized that distress may encourage PTG since both often coexist. However, authors also stated that high levels of PTG correlate with lower distress even though this is not always the case. In fact, post-traumatic stress disorder (PTSD) predicted PTG in a longitudinal study involving war veterans; even so, among PTSD symptoms only hyper-arousal was a PTG predictor over time while depression and anxiety were not (Dekel et al., 2012). More recently, a meta-analysis investigated the relationship between PTG and PTS in the oncological population finding a small but positive association between the two constructs. This overall outcome was characterized by a great heterogeneity among the included studies in relationship direction, magnitude and effect sizes; therefore, clear conclusions cannot be drawn about this issue. However, the PTS/PTG relationship appears to be stronger for advanced cancer patients and not affected by the PTG assessment instrument used or by the time since diagnosis (Casellas-Grau et al., 2017; Marziliano et al., 2020). Conversely, Ochoa et al. (2019) believe that the course of time is an important factor in facilitating a constructive stress-growth balance. The authors suggest that PTG interventions should be implemented after the conclusion of cancer treatments, when patients can accommodate their experience and appraise important changes. At this time, PTG interventions might encourage self-regulation, active coping and cognitive restructuring, while discouraging avoidance which is usually associated with PTS symptoms. Likewise, meaning making and relational harmony with significant others should be sustained.

The literature has highlighted a dearth of interventions targeting meaning making and PTG processes in cancer patients (Lee et al., 2004; Henoch and Danielson, 2009). A meta-analysis including randomized controlled trials (RCTs) of psychological interventions assessing PTG following traumatic experiences reported that PTG was never investigated as a primary outcome (Roepke, 2015). Nevertheless, among the non-RCT studies excluded from the meta-analysis, the author mentioned innovative interventions evaluating meaning and PTG as primary outcomes and reporting promising results with cancer patients (Garlan et al., 2011; Garlick et al., 2011). Among these, we appreciated the Expressive Writing (EW) intervention, which has been shown to increase meaning in life and trauma-related growth, and in a Romanian study to decrease depression in a sample of female cancer patients (Kállay and Băban, 2008).

Trauma and personal growth literature has suggested that writing can be effective if used to encourage not only emotional, but also a more adaptive narrative disclosure, consisting in a re-elaborated version of traumatic events such as a cancer diagnosis, triggering deliberate emotional and cognitive elaboration and contrasting automatic non-adaptive rumination (Triplett et al., 2012; Freda and Martino, 2015). This process would entail a two-fold path: on one hand, writing could have a cathartic purpose through which the person can release, clarify, and regulate his/her emotions (Lepore and Smyth, 2002; Pennebaker and Chung, 2007); on the other hand, it could prompt the meaning reconstruction process by means of a cognitive reappraisal of the cancer diagnosis and treatment, thus dissociating the event from the initial automatic emotional reaction.

EW is a technique implemented in order to promote psychological and physical well-being through the written expression of deep thoughts and emotions connected to an event (Pennebaker and Beall, 1986). Research has highlighted that EW often has positive consequences on health (Baikie and Wilhelm, 2005; Pennebaker and Chung, 2007). However, findings from studies on cancer patients on the efficacy of EW in reducing psychological symptoms and distress are not univocal (Jensen-Johansen et al., 2013; Merz et al., 2014; Zachariae and O’Toole, 2015). Considering that even little clinical effects of an inexpensive intervention can be significant, these findings highlight the need for further investigating the role of potential moderators, as well as diverse writing techniques in cancer patients.

The guided disclosure protocol (GDP), conceived by Duncan and Gidron (1999), is a writing intervention aiming to facilitate not only emotional disclosure but also cognitive processing of traumatic experiences. By guiding individuals to report in detail traumatic situations and to deliberate on past, present, and future impact of trauma, this protocol goes beyond the pure emotional disclosure of the original EW technique (Pennebaker and Beall, 1986; Arden-Close et al., 2013). The benefits of GDP have been documented by two studies. The first of these, performed by Gidron et al. (2002) on a sample of frequent clinic attenders, the experimental group participants showed lower symptom levels and made fewer clinic visits compared to controls. The second study was performed by Martino et al. (2013a) on parents of children treated for acute lymphoblastic leukemia. After the writing intervention, both psychological (e.g., anxiety, depression) and somatic symptoms were reduced in the experimental group.

Other authors have found no evidence for the effectiveness of GDP. This is the case of a study by Arden-Close et al. (2013) on the effect of GDP on perceived distress and quality of life in ovarian cancer couples. Their assignment involved writing about diagnosis and treatment for 15 min in three succeeding day sessions; however, the participants expressed a need for longer sessions. Moreover, despite the non-significant results, the intervention did buffer the distressing consequences of intrusive thoughts in participants from the experimental group. A plausible explanation for the lack of statistically significant effects on the main outcomes could be a too brief time gap among sessions. In fact, in a study aimed at decreasing PTSD symptoms in breast cancer patients, participants in the GDP group presented a significant decrease in intrusion and irritability compared to the control group receiving no treatment when the time gap between sessions was 15–20 days (Martino et al., 2013b).

To the best of our knowledge, no RCT has implemented the GDP in order to promote the meaning-making process aiming to foster positive changes as a consequence of experiencing a traumatic event like a cancer diagnosis. The majority of the studies that assess the relationship between meaning making and PTG are correlational, and there is a dearth of well-designed RCTs (Lee et al., 2004; Henoch and Danielson, 2009; Roepke et al., 2014). Since the GDP has been mainly employed for reducing PTSD symptoms (Arden-Close et al., 2013; Martino et al., 2013a,b), the present study investigates the hypothesis that the GDP promotes PTG through the process of meaning making.

In this randomized controlled trial, we compared GDP against a control intervention in stage I-III breast and colon cancer patients at the end of their adjuvant chemotherapy. These tumors were chosen for their high prevalence, good prognosis, and low risk of recurrence after the end of treatment. Thus, there can be room for these patients to benefit from interventions targeting potentially past traumatic events (e.g., diagnosis and treatment) through emotional processing and cognitive restructuring (e.g., GDP).

Patients were recruited between January 2016 and August 2020 during the follow-up clinical consultation. The general objective of this experimental study was to assess the efficacy of the GDP intervention in this population assessing outcomes within one month from the last writing session (T1 - i.e., 3 months from baseline) and at 4-month follow-up (T2 - i.e., 6 months from baseline).

The primary aim of the present RCT was to assess the efficacy of the Guided Disclosure Protocol (GDP) as compared to a generic writing intervention in promoting post-traumatic growth (PTG), measured with the total score of the Italian version of the Post-Traumatic Growth Inventory (PTGI) at the end of the adjuvant chemotherapy in patients with stage I-III breast or colon cancer.

The secondary aims were to assess the efficacy of the GDP as compared to a generic writing intervention at T1 and T2 in:

- Promoting the single dimensions assessed by the five factors of the PTGI: spiritual change, change in philosophy of life and self-conception, changes in relationships, discovery of new interests and values in life, and discovery of personal resources available for themselves and others;
- Enhancing subjective meaning as constructed during life-threatening illness, assessed with the total score of the Constructed Meaning Scale (CMS), and the two dimensions of the scale, i.e., disease as permanent damage and process of adaptation;
- Decreasing the distressing consequences of a traumatic event assessed by means of the total score of the Impact of Event Scale (IES) and the two dimensions of the scale, i.e., intrusive thoughts and avoidance of certain feelings, thoughts, or situations;
- Reducing overall emotional distress assessed with the total score of the Hospital Anxiety and Depression Scale (HADS), and the two dimensions of the scale: anxiety and depression.
- Investigating the relationship between post-traumatic growth and constructed meaning, testing the hypothesis that a change in PTG would be mediated by constructed meaning.

## 2 Materials and methods

### 2.1 Study design

This is a multicenter randomized controlled trial. Eligible patients were randomized to receive the guided disclosure protocol (experimental group) or a generic writing intervention (control group), and were assessed at baseline (T0 - before the intervention), after the conclusion of the intervention (T1 - at 3 months, ± 15 days, from baseline), and at 6 months, ± 15 days, from baseline (T2 - follow-up). More detailed information can be retrieved in the study protocol (Cafaro et al., 2019).

### 2.2 Population

Patients were assessed for their eligibility in the oncological settings of five Italian hospitals: Arcispedale Santa Maria Nuova, Reggio Emilia (the coordinating center); Guastalla Hospital, Reggio Emilia; Mariano Santo Hospital, Cosenza; Scientific Institute of Romagna for the Study and Treatment of Cancer (IRST), Meldola, Forlì; Sacro Cuore-Don Calabria Hospital, Negrar, Verona.

Patients aged 18 years or over, with native proficiency in written and spoken Italian and with a histologically confirmed stage I-III breast or colon cancer, who had completed adjuvant chemotherapy by no more than 8 months and were disease-free at the last follow-up visit, were considered eligible for the trial. Exclusion criteria included patients who had received a structured psychological intervention delivered by a psychologist or by a psychiatrist for at least 6 months, or a psychopharmacological treatment for a codified psychiatric disorder (according to the Diagnostic and Statistical Manual of Mental Disorders, Fifth Edition, DSM-5) during the last 3 years.

In each participating center, a research group composed of nurses and psychologists was established to perform patient recruitment and assessment. Eligible subjects were identified by oncologists during the follow-up consultation or by researchers through consulting the clinical records of potentially eligible patients. Oncologists provided eligible patients with basic information on the trial during their consultation.

Eligible patients who agreed to participate gave their written consent. Afterwards, researchers administered baseline questionnaires, contacted the trial center for randomization and explained the writing tasks to the participants according to the allocated condition.

### 2.3 Assessment

The socio-demographic and clinical characteristics of the patients were collected after enrollment in the study. The same booklet containing the questionnaires to be filled in by the patients was administered for self-assessment at baseline (T0), at 3 months ±15 days from baseline (T1), and at 6 months ±15 days from baseline (T2). The booklet contained four questionnaires to be filled in by the participants: the Italian versions of the Post-traumatic Growth Inventory (PTGI), the Constructed Meaning Scale (CMI), The Impact of Event Scale (IES), and the Hospital Anxiety and Depression Scale (HADS).

*The Post-traumatic Growth Inventory (PTGI)* (Prati and Pietrantoni, 2006) is a 21-item questionnaire assessing post-traumatic growth. Items are rated on a 6-point Likert scale (0 to 5), and patients are asked to what extent they have experienced a change in their lives as a result of their illness. The total score ranges from 0 to 105, where higher scores indicate higher levels of post-traumatic growth. Five sub-scales can be calculated: spiritual change (2 items, score 0–10), change in philosophy of life (7 items, score 0–35), change in relationships (5 items, score 0–25), new interests and values in life (3 items, score 0–15).

*The Constructed Meaning Scale (CMS)* (Fife, 1995; Giorgi et al., 2007) is an 8-item questionnaire assessing subjective meaning as it is constructed during life-threatening illness. Items are rated on a 4-point Likert scale (1 to 4). The total score ranges from 8 to 32, where higher scores indicate a higher sense of meaning. Two subscales can be calculated: disease as permanent damage (4 items, score 4–16) and process of adaptation (4 items, score 4–16).

*The Impact of Event Scale (IES)* (Horowitz et al., 1979; Pietrantonio et al., 2003) is a 15-item questionnaire measuring the distressing consequences of a traumatic event. Items are rated on a 4-point Likert scale (1 to 4) and the total score ranges from 15 to 60. Higher scores indicate higher levels of distress. Two subscales can be calculated: intrusive thoughts (8 items, score 8–32) and avoidance of certain feelings (4 items, score 4–16).

*The Hospital Anxiety and Depression Scale (HADS)* (Zigmond and Snaith, 1983; Costantini et al., 1999; Iani et al., 2014) is a 14-item questionnaire developed to measure emotional distress resulting from diagnosis of life-altering illness and treatment in hospital outpatient settings. The scale, widely used with cancer patients, showed high sensitivity and specificity in identifying anxiety and affective disorders (Grassi et al., 2009). Also, its two-factor structure was confirmed with Italian cancer patients (Annunziata et al., 2011). Items are rated on a 4-point Likert scale (0 to 3) and the total score ranges from 0 to 42. Higher scores indicate higher levels of distress. Two subscales can be calculated: anxiety (7 items, score 0–21) and depression (7 items, score 0–21).

Moreover, at baseline, the expectancy about the intervention was assessed by asking patients to indicate “… the extent to which you think that writing will help you to grow, finding new resources in yourself since the illness experience” (Boot et al., 2013). Responses are provided on a scale ranging between 1 (*not at all*) and 7 (*very much*).

Finally, patients were asked to evaluate their perception of the task difficulty on a 5-point rating scale (ranging from “*very easy, score = 1”* to “*very difficult, score = 5”*).

### 2.4 The interventions

#### 2.4.1 Experimental group

Patients randomized in the experimental group were asked to carry out the GDP intervention. It consisted of three 20-min writing sessions where participants were first asked to remember the facts related to their cancer illness chronologically (session 1), then to identify the emotions concerning those facts, appraise immediate changes in priorities, think about their current feelings and coping mechanisms they have learned (session 2). Lastly, they were asked to reflect on how the traumatic experience has changed their attitude toward life and themselves, teaching them to deal with other difficult situations/troubles which may arise in the future (session 3). The original GDP instructions were translated into Italian and adapted to the traumatic event of cancer experience for our study purpose (Cafaro et al., 2019; see Table 1).

**Table 1:** GDP instructions for each session.

Task for session 1	Describe memories concerning your cancer illness in chronological order, assuming a detached, impersonal, and objective attitude.
Task for session 2	Describe: (a) thoughts and emotions perceived during the illness experience; (b) the impact of the illness on your daily life, and how it has changed your attitudes toward life.
Task for session 3	Focus on your current situation, think about the entire illness experience, and report on the following aspects: Your present thoughts and feelings and how they differ from the ones you felt during the illness experience. The extent to which you have come to terms with, understand, and appreciate yourself for having dealt successfully with the illness. What you have learned from the illness in terms of personal insights, knowledge, and skills, and how these resources could be useful in the future. How you will cope with other similar events in the future.

Participants were asked to identify a quiet place and time in their home to attend the writing task. The first writing session had to be completed within two weeks after the initial assessment (T0), and the following two sessions once every two weeks.

#### 2.4.2 Control group

Patients randomized in the control group were asked to complete a generic writing intervention consisting of three 20-min writing sessions where they were prompted to write about events in their daily lives which happened during the previous week; they were also asked to focus on facts, assuming a detached, impersonal, and objective attitude. As for the experimental group, the participants were asked to find a quiet place and time to write. The same time interval had to be respected between sessions as for the GDP (Cafaro et al., 2019).

The day before each writing session, participants in both groups were contacted by telephone by the study coordinator (VC), who checked their understanding of the instructions reported in the intervention booklet and reminded them to attend to the writing task. Failure to contact the patient was recorded on the patient form. Participants were invited to hand over their writings to the researcher at the second assessment (T1) so that compliance with the task was also checked.

### 2.5 Ethical issues

The study protocol was approved by the Ethics Committee of the Reggio Emilia coordinating center (code no. 2016/0012561) and then by the Ethics Committee of the other participating centers: Meldola-FO (code no. 4562/2016 I.5/127), Cosenza (code no. 81, 15th July 2016), Negrar-VR (code no. 49997, 25th October 2016), and Guastalla (code no. 4228, 11th January 2018). The trial was registered with ClinicalTrials.gov (Protocol Record 2015/0024360).

### 2.6 Statistical methods

#### 2.6.1 Randomization

Eligible patients were randomly assigned to the GDP condition or to the control condition with an allocation ratio 1:1. Randomization was carried out through a central phone randomization center using computer generated random numbers. After the registration of each patient’s basic information, the trial center attributed a unique code to the included patient and communicated the allocated condition to the study coordinator.

#### 2.6.2 Statistical analyses

We estimated an effect size of 0.36 on the effect of psychological interventions on PTG, as a measure of effect of clinical interest, according to what was reported in a meta-analysis on the effect of psychological interventions on PTG (Roepke, 2015). In order to show an effect size of 0.36 with an alpha of 0.05 and a power of 0.80, a minimum of 123 subjects in both groups was estimated. Sample size was computed with a two-sided test using G power 3.1.3 (Faul et al., 2009).

To explore a potential imbalance in baseline characteristic between the two groups, a visual inspection was performed of the distribution of the patients’ demographic and clinical characteristics and of the distribution of the scores at baseline of the four scales.

According to the study protocol (Cafaro et al., 2019), we performed the primary analysis through a 2 × 3 mixed factorial design analysis of variance (ANOVA) to determine whether there was a significant change in PTG between the two groups at 3 and 9 months after baseline. The “between” factor was intervention with two levels (yes/no) and the “within” factor was time with three levels (T0/T1/T2). An interaction effect was evaluated to determine whether the intervention had a significant effect at 3 and 9 months after baseline compared to the control group.

The same analysis was repeated for the other four scales (PTGI, CMS, IES and HADS).

To consider differences in the distribution of the scores at baseline, a specific analysis not planned in the protocol was performed. We first estimated mean differences from baseline (T1 minus T0 and T2 minus T0) for each group. Subsequently we compared the two mean differences between the two groups by means of an independent t-test. For the PTGI and CMS scales, a positive mean difference meant better performance of the experimental group from baseline as compared to the control group. Conversely, for the IES and HADS scales, a positive mean difference meant increased distress in the experimental group from baseline as compared to the control group. For each mean difference 95% CI was estimated. *p*-value was reported only for the four scales.

## 3 Results

Between January 2016 and August 2020, recruitment was carried out at five participating cancer centers. One center did not track the number of patients who declined to participate in the study; in the other four centers, a total of 54 (39.7%) patients declined participation. Among those who accepted to participate in the study in all five centers, a total of 104 patients were randomized by the hospitals of Reggio Emilia (*n* = 47), Meldola (*n* = 22), Negrar (*n* = 15), Cosenza (*n* = 15), and Guastalla (*n* = 5). One participant withdrew consent, and one was excluded because of psychiatric drug use not ascertained at enrollment. Of the remaining 102 participants, 49 received the experimental intervention and 53 received the control intervention. Twenty-one patients dropped out of the study before the post-intervention assessment, 10 of whom from the intervention group and 11 from the control group. For three participants, dropout was due to problems in performing all three writing sessions because of family issues. Ten patients were unreachable for the questionnaire administration, one declined to return for assessment, one was excluded because of disease relapse, one was unable to return because of moving to a new house, one patient stated that she found it difficult to express herself in writing, one found the writing task not meaningful, and three had other health issues.

**Figure 1:**
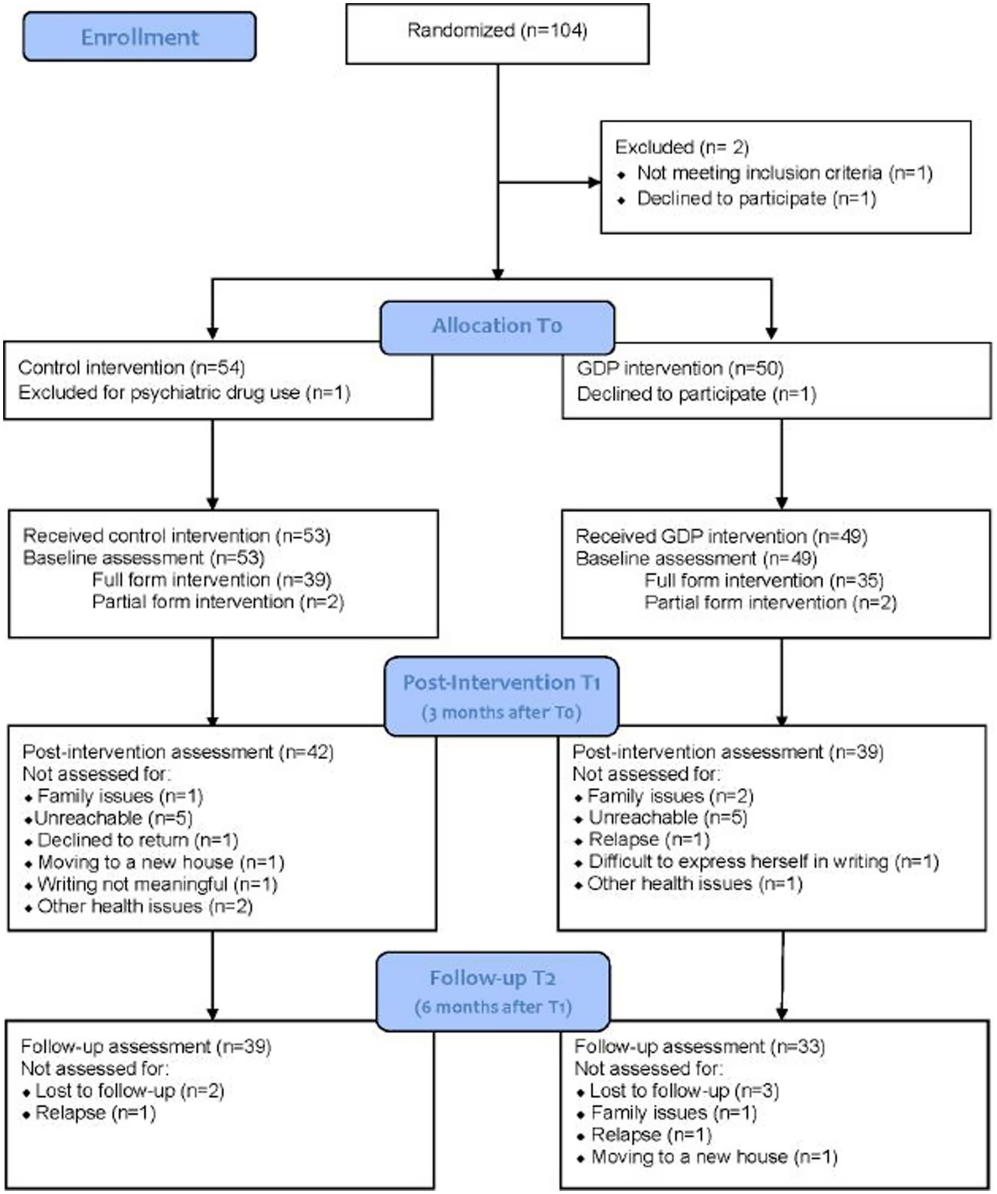
RCT flow diagram.

Complete data, including outcome evaluation at three time points (T0, T1 and T2), were available for 72 patients (33 for the experimental group and 39 for the control group). Fourteen patients dropped out before the follow-up assessment. Five were lost at follow-up, two had a disease relapse, one did not return due to family issues and one because of moving to a new house (see Figure 1).

The demographic and clinical characteristics of the 102 patients included in the analysis are reported in Table 2. No major differences were observed in the distribution of all variables between the two groups. Most were women (*n* = 81; 79.4%), with a mean age of 55.6. Seventy patients (68.6%) had breast cancer, 32 colon cancer, most of them with stage I (*n* = 28; 27.5%) or stage II disease (*n* = 45; 44.1). Forty-six participants (45.1%) had a high school diploma, and 72 (69.6%) had a partner. Time from the first diagnosis ranged from 1 to 27 months (mean = 13.1).

**Table 2:** Patient characteristics by treatment group at baseline.

	Control (*N* = 53)	GDP (*N* = 49)
*Sex – no. (%)*		
Females	45 (84.9)	36 (73.5)
Males	8 (15.1)	13 (26.5)
*Age*		
Mean (DS)	54.0 (11.1)	57.2 (10.4)
*Tumor – no. (%)*		
Breast	38 (71.7)	32 (65.3)
Colon	15 (28.3)	17 (34.7)
*Disease Stage – no. (%)*		
I	18 (34.0)	10 (20.4)
II	20 (37.7)	25 (51.0)
III	11 (20.8)	13 (26.5)
*Civil Status – no. (%)*		
Single	11 (20.8)	10 (20.4)
Married	34 (64.2)	37 (75.5)
Unknown	8 (15.1)	2 (4.1)
*Education – no. (%)*		
Primary School – Middle School	17 (32.1)	16 (32.7)
High School	27 (50.9)	19 (38.8)
Bachelor’s Degree	6 (11.3)	14 (28.6)
Unknown	3 (5.7)	−
*Compliance at assessment – no. (%)*		
T0	53 (100)	49 (100)
T1	42 (79.2)	39 (79.6)
T2	39 (73.6)	33 (67.3)

Most of the patients in the GDP group who completed the T1 assessment evaluated the intervention as “quite easy” (*n* = 10; 20.4%) or “not easy or difficult” (*n* = 10; 20.4%). Other patients evaluated the writing task as “very easy” (*n* = 7; 14.3%) and “quite difficult” (*n* = 3; 6.1%). Nineteen participants did not evaluate the intervention (38.8%).

A minimal but significant higher expectancy (*p*-value = 0.03) about the intervention was observed in the control group (mean = 4.94; SD = 1.3) as compared to the experimental group (mean = 4.35; SD = 1.5).

A visual inspection of the distribution of the scores at baseline (Table 3) of the four analyzed scales showed higher levels of post-traumatic growth in the control group as compared to the experimental group (PTGI mean = 50.5 vs. 40.7, respectively) and in psychological distress (HADS mean = 8.9 vs. 6.8, respectively). Similar differences were observed for the subscales of the two questionnaires.

**Table 3:** Outcome assessment at the three planned evaluations.

	T0		T1		T2	
	Control (*N* = 53)	GDP (*N* = 49)	Control (*N* = 42)	GDP (*N* = 39)	Control (*N* = 39)	GDP (*N* = 33)
	Mean (SD) median	Mean (SD) median	Mean (SD) median	Mean (SD) median	Mean (SD) median	Mean (SD) median
PTGI	50.5 (25.5) 46.0	40.7 (21.5) 41.0	51.5 (25.4) 55.5	48.3 (23.4) 47.0	51.1 (29.3) 63.0	47.2 (23.7) 53.0
Relating to others	16.8 (10.3) 18.0	14.7 (7.9) 15.0	17.0 (9.9) 19.5	16.6 (9.2) 17.0	16.7 (10.5) 21.0	15.9 (8.8) 16.0
New possibilities	10.3 (7.0) 9.0	7.7 (5.4) 8.0	10.7 (6.8) 11.5	9.6 (6.3) 9.0	10.8 (7.4) 13.0	9.7 (6.6) 10.0
Personal strength	11.4 (5.9) 12.0	9.4 (6.0) 9.0	11.6 (5.4) 13.5	10.8 (5.6) 12.0	11.3 (6.0) 13.0	11.2 (5.3) 12.0
Spiritual change	2.6 (3.3) 1.0	1.2 (2.1) 0.0	3.1 (3.4) 2.0	1.9 (2.4) 0.0	2.9 (3.5) 1.0	1.8 (2.1) 1.0
Appreciation of life	9.4 (4.0) 4.0	7.8 (4.5) 8.0	9.2 (4.4) 10.0	9.3 (4.3) 11.0	9.4 (5.1) 11.0	8.7 (4.8) 9.0
CMS	18.9 (3.3) 19.0	19.1 (2.7) 19.0	19.0 (2.3) 19.0	19.5 (2.2) 20.0	19.5 (2.1) 19.0	19.3 (2.0) 19.0
Process of adaptation	11.4 (2.8) 12.0	12.1 (2.2) 12.0	11.5 (2.4) 12.0	12.1 (2.0) 12.0	11.9 (2.4) 12.0	12.1 (2.0) 12.0
Permanent damage	7.5 (2.7) 7.0	7.1 (2.2) 7.0	7.5 (2.3) 8.0	7.4 (2.2) 7.0	7.5 (2.5) 7.0	7.2 (2.4) 7.0
IES	30.2 (10) 30.0	28.8 (8.6) 29.0	30.7 (9.1) 31.0	30.7 (9.1) 31.0	30.4 (9.8) 29.0	30.7 (9.8) 29.0
Intrusion	22.9 (8.0) 22.0	21.5 (6.1) 22.0	21.1 (6.1) 21.5	22.5 (6.2) 22.0	22.5 (7.1) 22.0	22.4 (7.0) 21.0
Avoidance	7.2 (2.9) 7.0	7.2 (3.3) 7.0	7.5 (3.1) 7.0	8.3 (3.8) 8.0	7.8 (3.6) 7.0	8.2 (3.7) 7.0
HADS	8.9 (7.4) 7.0	6.8 (5.8) 5.0	8.1 (5.9) 7.0	7.6 (6.9) 5.0	6.9 (5.5) 5.0	7.4 (6.4) 6.0
Anxiety	5.5 (4.8) 5.0	4.5 (3.7) 4.0	5.0 (4.0) 5.0	4.9 (4.2) 4.0	4.4 (3.7)4.0	5.1 (4.0) 5.0
Depression	3.4 (3.4) 2.0	2.3 (2.7) 2.0	3.0 (2.5) 3.0	2.7 (3.2) 2.0	2.5 (2.4) 2.0	2.3 (3.1) 1.0

PTGI, Post-traumatic Growth Inventory; CMS, Constructed Meaning Scale; IES, Impact of Event Scale; HADS, Hospital Anxiety and Depression Scale.

SD, Standard Deviation; GDP, the experimental group who received the guided disclosure protocol.

Independent sample *t*-tests were conducted to compare dependent variables (PTG, CMS, IES, HADS) in control and GDP groups.

Following the primary statistical analysis, a mixed factorial design ANOVA was performed on the PTGI to investigate differences between the experimental and control groups at T0, T1 and T2. The analysis revealed that there was not a statistically significant interaction between the effect of type of intervention and time point, *F*(1.795, 125.622) = 1.151, *p* = 0.315. Simple main effect analysis showed that time did not have a statistically significant effect on post-traumatic growth, *F*(1.795, 125.622) = 0.635, *p* = 0.515. Lastly, simple main effect analysis showed that the type of intervention did not have a statistically significant effect on post-traumatic growth, *F*(1.70) = 0.489, *p* = 0.487.

A positive effect of the intervention, although not statistically significant, was observed at T1 in terms of post-traumatic growth (PTGI mean difference = −5.8; 95% CIs = −14 to 2.4; *p* = 0.166), and distress after a traumatic event (IES mean differences = −2.2; 95% CIs = −5.0 to 0.7; *p* = 0.142). No major differences were observed for the other scales at T1 and for all scales at T2. Namely, no statistically significant differences were observed between groups in the PTGI five factors (spiritual change, change in philosophy of life and self-conception, changes in relationships, discovery of new interests and values in life, and discovery of personal resources available for themselves and others), in the CMS and its dimensions (disease as permanent damage and process of adaptation), in the two dimensions of the IES (intrusive thoughts and avoidance) and in the HADS and its two subscales (anxiety and depression). Finally, because of the lack of statistical significance of the outcomes, it was not feasible to test the hypothesis that a change in PTG would be mediated by constructed meaning (Table 4).

**Table 4:** Mean differences (T1 or T2 minus T0) between the experimental and control groups.

	Control T1-T0 (*N* = 42)	GDP T1-T0 (*N* = 39)	Mean difference (95% CI)	*p*-value	Control T2-T0 (*N* = 39)	GDP T2-T0 (*N* = 33)	Mean difference (95% CI)	*p*-value
	Mean	Mean			Mean	Mean		
PTGI	−0.6	5.2	−5.8 (−14–2.4)	0.166	−1.3	2.1	−3.4 (−13–7.2)	0.526
Relating to others	−0.3	1.5	−1.8 (−5.1–1.6)		−0.5	0.2	−0.7 (−4.5–3.0)	
New possibilities	−0.2	1.2	−1.4 (−4.1–1.2)		−0.1	0.8	−0.8 (−3.7–2.0)	
Personal strength	−0.2	1.1	−1.3 (−3.3–0.6)		−0.6	1.0	−1.6 (−4.2–1.0)	
Spiritual change	0.4	0.6	−0.1 (−1.1–0.8)		0.2	0.5	0.3 (−1.6–0.9)	
Appreciation of life	−0.3	0.8	−1.1 (−2.5–0.3)		−0.2	−0.4	0.2 (−1.8–2.1)	
CMS	−0.1	0.4	−0.4 (−1.9–1.0)	0.548	0.5	0.2	0.2 (−1.3–1.7)	0.770
Process of adaptation	−0.1	0.3	−0.4 (−1.4–0.6)		0.1	0.4	−0.3 (1.2–0.7)	
Permanent damage	0.1	0.1	−0.1 (−0.9–0.9)		0.4	−0.1	0.5 (−0.8–1.7)	
IES	−1.0	1.2	−2.2 (−5.0–0.7)	0.142	1.0	0.6	0.3 (−3.9–4.5)	0.882
Intrusion	−1.4	0.3	−1.7 (−3.8–0.4)		0.1	0.1	0.1 (−3.1–3.3)	
Avoidance	0.5	0.9	−0.5 (−1.7–0.8)		0.8	0.6	0.2 (−1.3–1.7)	
HADS	0.1	0.3	−0.1 (−2.3–2.0)	0.916	−0.7	0.3	−1.0 (−3.6–1.7)	0.465
Anxiety	0.3	0.1	0.2 (−1.0–1.4)		−0.1	0.3	−0.4 (−2.0–1.2)	
Depression	−0.1	0.2	−0.3 (−1.5–0.9)		−0.6	−0.1	−0.6 (−1.9–0.7)	

*p*-values estimated via a t-test for independent samples.

Mean difference is the difference between the means of the GDP group (T1 or T2 minus T0) minus the means of the control group (T1 or T2 minus T0). For the PTGI and CMS scales, a positive mean difference means better performance in the experimental group from baseline as compared to the control group. Conversely, for the IES and HADS scales, a positive mean difference means increased distress in the experimental group from baseline as compared to the control group.

PTGI: Post-traumatic Growth Inventory, CMS: Constructed Meaning Scale, IES: Impact of Event Scale, HADS: Hospital Anxiety and Depression Scale. GDP is the group who received the guided disclosure protocol.

## 4 Discussion

### 4.1 Summary of findings

This study intended to fill the gap in the literature concerning the lack of RCTs with the primary aim of assessing PTG in cancer patients through a writing intervention. The guided disclosure protocol (Duncan and Gidron, 1999) was chosen because of its specific focus on both emotional expression and cognitive processing of a traumatic event, leading therefore to a global reorganization of the illness experience. The core motivation leading to the trial design and implementation was to trigger in off-therapy cancer patients a virtuous loop enhancing personal strength and resilience, renovating beliefs and priorities and life appreciation.

Notwithstanding the rigorous design of the study, our findings do not reach statistical significance for any of the proposed aims. Consequently, it was not possible to test the mediation hypothesis that constructed meaning would lead to PTG (Cafaro et al., 2019).

The non-significance of the trial results does not necessarily imply that the study hypotheses should not deserve further consideration. As reported in the study protocol (Cafaro et al., 2019), a sample size of minimum 246 participants (123 per group) was required to detect the intervention effect, and unfortunately a total sample of only 104 patients were enrolled in this study. Despite the lack of statistical power of the trial, the mean difference in PTG at T1 shows a positive effect of the GDP intervention as compared with control intervention (see Table 3). In the authors’ opinion, the trend of the data might indicate that, with a larger sample, the experimental group could have significantly increased PTG compared to the control group. Besides the possible effect of the experimental intervention on PTG, data appear to show the tendency to an increase of distress as measured with the total score of the Impact of Events Scale in the GDP group. Lack of significance still do not allow to draw any conclusions, however this trend might boost the hypothesis that PTG and PTS are closely interrelated even though further research is warranted in order to determine the nature of their connection (e.g., Tedeschi and Calhoun, 2004; Dekel et al., 2012).

### 4.2 Recruitment difficulties in the RCT

Adequate sample size was not reached for a number of issues concerning recruitment. In some participating centers, oncologists had difficulty selecting eligible patients and presenting the study to them within the strict timeframe of the medical consultation. For example, some medical consultations were scheduled more than 8 months after the end of chemotherapy and therefore exceeded the inclusion criteria time window. Additionally, hospital measures taken to contain the COVID-19 pandemic led to recruitment interruption from the end of February 2020. In light of these problems, six months later the coordinating center research group decided to close recruitment before reaching the planned sample size, at 4 years and 7 months from the beginning of the study.

Recruitment difficulties in RCTs are well-known, and several studies have investigated the reason underlying this issue. Among the recruitment problems common to every RCT, Howard et al. (2009) mentioned several concerning clinicians involved as recruiters, including misconceptions about RCTs, difficulties in maintaining equipoise between study arms, and different interpretations of eligibility criteria. Other recruitment issues involve both recruiters and participants and concern motivation to participate, the burden of the study, and continuity of participation between planning, recruitment and beginning of the research (Axén et al., 2021). Within our study, some strategies were employed in order to overcome these barriers. In each participating center, a research group composed of nurses and psychologists was designated to carry out patient recruitment and assessment. Oncologists involved in the study were trained by the research group about the study design, the patient eligibility criteria, and the collaboration requested of them as clinicians. In some of the centers, the day before follow-up medical consultations, the researchers checked each paper medical record to assess the potential eligibility of patients scheduled for a follow-up visit. Consequently, the burden on oncologists related to the study was reduced by limiting their involvement in checking the eligibility of patients initially identified by the researchers according to clinical criteria, and by briefly introducing the study to them. Patients who expressed interest in the study were then approached by researchers, who verified their eligibility according to the other criteria, provided them with detailed information and, in case of acceptance to participate, managed the subsequent phases of the assessment procedures. Training for researchers was planned and implemented by the coordinating center research group, who also developed a user manual providing a detailed description of each phase of the study, including not only recruitment, randomization and assessment procedures but also skills for approaching eligible subjects, explaining the collaboration required of them, and coping with different communicative scenarios. Although these strategies ensured the methodological rigor of the study, they were very time consuming and resulted in slowing down the enrollment process. In order to overcome these obstacles, trials should make use of the stable and continuous presence of one researcher entirely dedicated to the study, with the task of supporting professionals involved at each time point of the study process, as also highlighted by Aspden et al. (2021) in their qualitative study on views of healthcare professionals concerning recruitment in psychosocial RCTs. Lastly, in our trial, at the beginning of the recruitment, electronic medical records were still not in use within the participating centers; their increasing employment in the last few years could facilitate and accelerate the retrieval of patient information for future studies.

### 4.3 Feasibility and acceptability of the GDP intervention and assessment

Despite the above-mentioned recruitment issues and the high refusal rate of the study, data on compliance of the enrolled subjects throughout the study highlight the feasibility and acceptability of both the intervention and the assessment. Indeed, as the participants were requested to complete a writing task over six weeks and undergo three assessment sessions, the study registered a low drop-out rate. Among the reasons given by participants who dropped out of the study, only two cases were related to the task itself (i.e., writing not considered meaningful and difficulty in written expression). Each enrolled subject was followed by the study coordinator who, before every writing session, reminded him/her by phone about the task and responded to any doubts they expressed. Assessment was performed for each participant by the same researcher who made the recruitment. This strategy reflects a rigorous attention to the entire trial process and is corroborated in the literature (Huang et al., 2018; Axén et al., 2021). The writing task was evaluated by most of the participants as “quite easy,” “not easy or difficult” and “very easy.” Therefore, the GDP seemed to be appreciated by the participants, who did not perceive the intervention as a burden.

### 4.4 Study contextual factors

The coordinating center recruited the majority of patients, which may be due to both different resource allocation in terms of personnel involved and shortcomings in monitoring the performance of the other participating centers. In fact, although specific training and a user manual were provided to them (including a sheet requesting information about the reasons for declining participation), a schedule for regular meetings to discuss recruitment successes and failures, as recommended by the methodological literature (Huang et al., 2018), was not included in the plans. Moreover, since the study did not have any financial support, the healthcare personnel participated in the study on a voluntary basis.

The recruitment problems encountered in this trial may be also due to the tendency, within the participating centers, to prioritize medical trials over studies on interventions concerning psychological aspects, an issue already documented in the literature (Aspden et al., 2021). As oncologists are often intensely engaged in the medical aspects of the disease, it is plausible that their interest and efforts are mainly focused on pharmacological studies. In our opinion, given the cultural shift toward developing integrated pathways in cancer care, promoting greater awareness of the role of interventions that target well-being and positive life change, in addition to curing the disease, is paramount.

## 5 Conclusion and future perspectives

As mentioned above, the findings from this study did not reach statistical significance for any of the planned outcomes. Nonetheless, as GDP is a promising intervention in promoting post-traumatic growth, and the lack of statistical significance may be due to the study being underpowered, we believe that this trial should be replicated paying particular attention to both the lessons learned from the issues that arose and to the strategies herein discussed to cope with such issues. Among these, presiding over and supporting recruitment, and closely monitoring the activity of participating centers throughout the study could be crucial. Moreover, a study with an adequate sample size could shed light on the factors that can mediate post-traumatic growth, as originally hypothesized in our study protocol (Cafaro et al., 2019). As also highlighted elsewhere (Tomich and Helgeson, 2004) these factors, together with other psychological variables that might influence both PTG and PTS and their relationship, should be investigated by further research. According to the literature, previous traumatic events, coping strategies, and attachment style in cancer patients may have an impact on post-traumatic outcomes (Cordova et al., 2017; Romeo et al., 2019). Studies involving such variables will provide researchers with information concerning which type of patients and when they may need and benefit more from psychological interventions such as GDP, aimed at increasing positive changes after the illness experience.

## Data availability statement

The datasets presented in this article are not readily available because consent for open data sharing was not included when participants provided informed consent. Requests for information should be directed to VC.

## Ethics statement

The studies involving humans were approved by Comitato Etico dell’Area Vasta Emilia Nord. The studies were conducted in accordance with the local legislation and institutional requirements. The participants provided their written informed consent to participate in this study.

## Author contributions

VC: Conceptualization, Data curation, Methodology, Project administration, Writing – original draft, Writing – review & editing. ER: Investigation, Writing – review & editing. GA: Data curation, Investigation, Writing – original draft. MC: Conceptualization, Methodology, Project administration, Resources, Supervision, Writing – original draft. FDV: Investigation, Writing – original draft. FF: Data curation, Software, Supervision, Writing – review & editing. SC: Conceptualization, Data curation, Methodology, Supervision, Writing – original draft. TB: Investigation, Writing – review & editing. GD: Investigation, Writing – review & editing. AP: Investigation, Writing – review & editing. LC: Investigation, Writing – review & editing. SDP: Investigation, Writing – review & editing. SP: Investigation, Writing – review & editing. MDI: Investigation, Writing – review & editing. GF: Investigation, Writing – review & editing. SDL: Conceptualization, Formal analysis, Investigation, Methodology, Resources, Supervision, Writing – original draft, Writing – review & editing.
